# The extremophile *Picrophilus torridus* carries a DNA adenine methylase M.PtoI that is part of a Type I restriction-modification system

**DOI:** 10.3389/fmicb.2023.1126750

**Published:** 2023-03-15

**Authors:** Pallavi Gulati, Ashish Singh, Manisha Goel, Swati Saha

**Affiliations:** ^1^Department of Microbiology, University of Delhi South Campus, New Delhi, India; ^2^Department of Biophysics, University of Delhi South Campus, New Delhi, India

**Keywords:** DNA adenine methylase, Type I restriction-modification system, modification methylase, *Picrophilus torridus*, acidophile, extremophile

## Abstract

DNA methylation events mediated by orphan methyltransferases modulate various cellular processes like replication, repair and transcription. Bacteria and archaea also harbor DNA methyltransferases that are part of restriction-modification systems, which serve to protect the host genome from being cleaved by the cognate restriction enzyme. While DNA methylation has been exhaustively investigated in bacteria it remains poorly understood in archaea. *Picrophilus torridus* is a euryarchaeon that can thrive under conditions of extremely low pH (0.7), and thus far no reports have been published regarding DNA methylation in this extremophile. This study reports the first experimentation examining DNA methylation in *P. torridus*. We find the genome to carry methylated adenine (m6A) but not methylated cytosine (m5C) residues. The m6A modification is absent at GATC sites, indicating the absence of an active Dam methylase even though the *dam* gene has been annotated in the genome sequence. Two other methylases have also been annotated in the *P. torridus* genome sequence. One of these is a part of a Type I restriction-modification system. Considering that all Type I modification methylases characterized to date target adenine residues, the modification methylase of this Type I system has been examined. The genes encoding the S subunit (that is responsible for DNA recognition) and M subunit (that is responsible for DNA methylation) have been cloned and the recombinant protein purified from *E.coli*, and regions involved in M-S interactions have been identified. The M.PtoI enzyme harbors all the motifs that typify Type I modification methylases, and displays robust adenine methylation in *in vitro* assays under a variety of conditions. Interestingly, magnesium is essential for enzyme activity. The enzyme displays substrate inhibition at higher concentrations of AdoMet. Mutational analyses reveal that Motif I plays a role in AdoMet binding, and Motif IV is critical for methylation activity. The data presented here lays the foundation for further research in the area of DNA methylation and restriction-modification research in this most unusual microorganism.

## 1. Introduction

DNA methylation typically occurs at adenine and cytosine residues. While methylation at adenine occurs exocyclically at the sixth position (N^6^-methyladenine: m6A), methylation at cytosine occurs either exocyclically at the fourth position (N^4^-methylcytosine: m4C), or endocyclically at the fifth position (5-methylcytosine: m5C). The primary DNA methylation mark in eukaryotes is m5C, while in bacteria the m6A methylation mark is the most widely prevalent. The marking of DNA by methylation has two distinctive roles: one, it modulates DNA interactions with proteins, thereby having an impact on DNA replication, DNA mismatch repair and gene expression, and second, it protects the bacterial and archaeal genome from self-destruction by their own restriction systems (Pingoud et al., 2014; Anton and Roberts, 2021).

The enzymes that mediate DNA methylation in bacteria and archaea use S-adenosyl methionine (AdoMet) as the methyl-group donor and are either orphan methyltransferases or a component of restriction-modification systems. Orphan methyltransferases (which do not have cognate restriction endonucleases) are associated with methylation events that regulate DNA-related transactions like replication and transcription. The Dam methylase which targets adenine methylation in the sequence 5′-GATC-3′ is the best known of these (Adhikari and Curtis, 2016). Methylases that are a component of restriction-modification (R-M) systems are designed to protect the genome from being attacked by cognate restriction endonucleases harbored by the organism. R-M systems are linked to the cell’s defense against incoming DNA, with the restriction endonuclease (RE) cleaving the foreign DNA that is not methylated at its specific recognition sequence. The host genome remains resistant to cleavage due to the recognition sites on the genome being methylated by the cognate modification methylase (MTase). Such methyltransferases can be a component of three types of R-M systems: Types I-III (Loenen et al., 2014b).

The Types I-III R-M systems differ in their subunit structure and organization, recognition sequence and cleavage site, and cofactor requirements. In the widely found Type II R-M systems, the modification methylase and the restriction endonuclease functions reside in independent enzymes that recognize the same palindromic sequence, with the endonuclease cleaving DNA within the (non-methylated) recognition sequence or just beyond. Type I and Type III R-M systems are complex multisubunit enzymes, with the modification methylase and restriction endonuclease functions residing in the same enzyme albeit different subunits (Rao et al., 2014; Loenen et al., 2014a). Type III R-M systems comprise two subunits: Mod and Res. The Mod is responsible for the recognition of the short asymmetric recognition sequence on DNA as well as its methylation, while the Res mediates DNA cleavage 25–27 bp downstream of the site. The Type I R-M systems comprise three subunits: S, M, and R, typically existing as pentamers of 1S, 2M, and 2R subunits. They recognize a specific bipartite DNA sequence *via* their S (specificity) subunit, modify the DNA at the recognition sequence *via* their M (modification) subunits, and cleave DNA through their R (restriction) subunits hundreds of base pairs away. While the restriction endonuclease exists in active form only as part of the multisubunit 1S2M2R enzyme, the modification methylase can also exist in active form independent of the R subunits, as a complex with the S subunit which it depends on for DNA sequence recognition (M2S1).

While DNA methylation and its effects have been extensively investigated in bacteria and eukaryotes, it remains a poorly understood area in archaea. The primary methylation marks in archaea are m6A and m4C, with m5C remaining undetected so far in these organisms. Single molecule real time (SMRT) sequencing has led to the analysis of the DNA methylomes of several bacteria and archaea. In a landmark effort, Blow et al. (2016) analyzed the DNA methylomes of 230 bacterial and archaeal species. Their study identified over 800 methylated motifs, emphasizing the extent of diversity among these groups of organisms (Blow et al., 2016). Other studies include the analysis of the DNA methylome of the hyperthermophilic archaeon *Thermococcus onnurineus* where over 2,000 sites of m6A were detected in addition to several sites of m4C (Lee et al., 2016), and the identification of DNA methylation at over 99% of the motifs 5′-GATC-3′ and 5′-AGCT-3′ in the genome of the marine thaumarchaeote *Candidatus Nitrosomarinus catalina* SPOT01 at the adenine and cytosine, respectively (Ahlgren et al., 2017). The DNA methylome of the hyperthermoacidophile *Sulfolobus acidocaldarius* was found to carry the m6A mark in the 5′-GATC-3′ motif occurring in two different genome contexts: 5′-AGATCC-3′ and 5′-GGATCT/C-3′ (Couturier and Lindas, 2018), and the m4C mark was also detected at the 5′-GGCC-3′ motif that was previously identified as the target site of the SuaI/M.SuaI restriction modification system (Prangishvili et al., 1985; Grogan, 2003).

Several R-M systems have been annotated in archaeal genomes but very few have been characterized. Among the earliest to be purified and studied were the Type II restriction endonuclease ThaI from *Thermoplasma acidophilum* (McConnell et al., 1978), three Type II restriction endonucleases from *Methanococcus aeolicus* (Schmid et al., 1984), a Type II R-M system from *Methanobacterium wolfei* (Lunnen et al., 1989), three Type II R-M systems from *Methanobacterium thermoformicicum* strains (Nolling and de Vos, 1992a,b), the PspG1 Type II R-M system from a *Pyrococcus* species (Morgan et al., 1998; Pingoud et al., 2003) and the SuaI/M.SuaI Type II R-M system in *Sulfolobus acidocaldarius* (Prangishvili et al., 1985; Grogan, 2003). Other Type II R-M systems that have been studied in archaea are PabI/M.PabI in *Pyrococcus abyssi* (Ishikawa et al., 2005; Watanabe et al., 2006) a type II restriction endonuclease SuiI from *Sulfolobus islandicus* (Suzuki and Kurosawa, 2016) and two Type IIG R-M systems TkoI and TkoII in *Thermococcus kodakarensis* (Zatopek et al., 2021). The only Type I R-M system studied in archaea to date is in *Haloferax volcanii* (Ouellette et al., 2018).

The present study was initiated with the aim of examining DNA methylation in the thermoacidophilic archaeon *Picrophilus torridus*. Originally isolated from a solfataric field in northern Japan, the *Picrophilus* species are the most acidophilic organisms isolated to date, growing optimally at a pH of 0.7 at 55–60°C, with an intracellular pH of ~4.6 (Schleper et al., 1995). When the 1.55 Mb genome of *Picrophilus torridus* was sequenced (Futterer et al., 2004) it was found to be AT-rich (64%) even though 92% of the genome was coding sequence. Our study found that the genome carried m6A methylation, but Dam-mediated methylation was not detectable. The investigation was taken forward with the examination of the modification methylase of the Type I R-M system annotated in its genome sequence, as all Type I R-M systems characterized to date demonstrate m6A methylation activity. The recombinant protein (M.PtoI) was found to mediate DNA adenine methylation under a wide range of conditions *in vitro,* and residues critical to methylation activity were identified.

## 2. Materials and methods

### 2.1. *Picrophilus torridus* cultures and genomic DNA isolation

*Picrophilus torridus* (strain DSM 9790) was cultured as described earlier (Arora et al., 2014). Briefly, cells were grown at 55°C in liquid culture with aeration, in medium of pH 1.0 comprising yeast extract (0.2%, Difco, United States), glucose (1%, Sigma, United States), potassium dihydrogen phosphate (0.3%), magnesium sulphate (0.05%), calcium chloride (0.025%) and ammonium sulphate (0.02%). For growth analysis, cultures were initiated with 1% (v/v) inoculum in media of different pH, from a starter culture at pH 1.0 that was at OD_600_ ~ 1.5. Growth was monitored by measuring OD_600_ every 24 h over a period of 14 days. Three experiments were set up in parallel and the average values of the three are plotted graphically, with error bars representing standard deviation. Genomic DNA was isolated from exponentially growing *Picrophilus torridus* cultures as described earlier (Arora et al., 2014). Briefly, harvested cells were resuspended in TEN buffer [20 mM Tris-Cl (pH 8.0), 1 mM EDTA, 100 mM NaCl], lysed by the addition of sodium lauroylsarcosine (1.6%) and Triton X-100 (0.12%), the lysate extracted with phenol, and genomic DNA precipitated using ethanol.

### 2.2. Cloning of M.PtoI genes

The ~1.3 kb gene encoding the S subunit was amplified off *Picrophilus torridus* genomic DNA using primers PtS-F (5′-TAAGATCTCATGAATAAAAAGGATTATAAT-3′) and PtS-R (5′-TACTCGAGATCACCACTTATTTTCAC-3′), with the proof-reading enzyme Phusion DNA polymerase (NEB, United States). The amplicon was cloned into pUC19, followed by subcloning into the BglII-XhoI sites of the pETDuet-1 vector (Novagen, United States), thus obtaining the pET-Duet/PtS clone. The ~1.7 kb gene encoding the M subunit was similarly amplified using the primers PtM-F (5′-TAGAATTCGATGGCAGATTTAAACTGG-3′) and PtM-R (5′-TAGCGGCCGCTTC ATAGTAACCAAG-3′), the amplicon cloned into pUC19, and then subcloned into the EcoRI-NotI sites of pET-Duet and pET-Duet/PtS, thus obtaining plasmids pET-Duet/PtM and pET-Duet/PtSM, respectively.

### 2.3. Creation of PtM mutants

All mutageneses were carried out through PCR using Phusion DNA polymerase. For creating PtM mutants the plasmid pUC/PtM was used as template. The C-terminal deletion mutant was created by PCR using the PtM-F primer in combination with PtM_Δ537-576_-R (5′-TAGCGGCCGCTTTATTGTCTATATATAAAG-3′). The ~1.6 kb amplicon thus obtained was cloned into pJET vector followed by subcloning into the EcoRI-NotI sites of pETDuet/PtS, yielding the plasmid pETDuet/PtSM_Δ537-576_. The NPPW and xxGxxG mutants were created by overlap PCR. To mutate the NPPW motif, the N-terminal part of the gene was amplified using primers PtM-F and PtM-N360A-W363A-R (5′-CTGGTTCGCTGGAGGCGCCG CAAC-3′) while the C-terminal part of the gene was amplified using primers PtM-N360A-W363A-F (5′-GTTGCGGCGCCTCCAGCGAACCAG-3′) and PtM-R. The full length amplicon obtained by amplification with PtM-F and PtM-R primers was cloned into pJET vector followed by subcloning into the EcoRI-NotI sites of pETDuet/PtS, yielding the plasmid pETDuet/PtSM_N360A-W363A_. To mutate the xxGxxG motif, the N-terminal part of the gene was amplified using primers PtM-F and PtM-G284S-R (5′-CCTGCAGTTGAACATGCCGG-3′) while the C-terminal part of the gene was amplified using primers PtM-G284S-F (5′-CCGGCATGTTCAACTGCAGG-3′) and PtM-R. The full length amplicon obtained using primers PtM-F and PtM-R was cloned into pJET vector followed by subcloning into the EcoRI-NotI sites of pETDuet/PtS, yielding the plasmid pETDuet/PtSM_G284S_.

### 2.4. Creation of PtS mutant

M.PtoI/S_211-222(A-G)_ was created using Overlap PCR. The N-terminal fragment of the mutant amplicon was produced using primers PtS-F and PtS_211-222(A-G)_-R (5′-GGCCCCGGCCC CAGCTCCACCTGCCCCTGCTCCTGCCAACTGTATTTCCTT-3′) and the C-terminal fragment of the mutant amplicon was produced using primers PtS_211-222(A-G)_-F (5′-GCAGGAGCAGGGGCAGGTGGAGCTGGGGCCGGGGCCTTCTGAAGGCATC-3′) and PtS-R. The full-length mutated gene was amplified using PtS-F and PtS-R and ligated into the BglII-XhoI sites of pET-Duet vector to create plasmid pET-Duet/PtS_211-222(A-G)_. This was followed by cloning the PtM gene into the EcoRI-NotI sites of pETDuet/PtS_211-222(A-G)_ to create plasmid pETDuet/PtS_211-222(A-G)_M.

### 2.5. Expression and purification of recombinant M.PtoI

The M.PtoI enzymes were expressed in *E.coli* BL21 Codon Plus cells (Stratagene, United States) that had been transformed with their pET-Duet clones. Expression of the wild type or mutant proteins was induced in cells growing exponentially (OD_600_ = 0.6–0.8) at 37°C using 1 mM IPTG. Cells were allowed to grow for a further 3 h at 37°C before harvesting by centrifugation and resuspension in lysis buffer (100 mM Tris.Cl pH8, 150 mM NaCl, 10% glycerol) containing protease inhibitors and lysozyme. This was followed by incubation of the cell suspension on ice for 20 min and lysis by sonication. After clarification of the lysates by high speed centrifugation they were subjected to cobalt-affinity chromatography using TALON metal affinity resin (Clonetech). The lysates were loaded on the column resin, the column extensively washed (with 100 mM Tris.Cl (pH 8), 1 M NaCl, 10% glycerol), and the bound proteins eluted using 100 mM Tris.Cl (pH 8), 250 mM imidazole, 10% glycerol. The M subunit of M.PtoI was similarly purified from bacteria transformed with pET-Duet/PtM.

### 2.6. *Picrophilus torridus* genomic DNA methylation analysis

Dot blot assay was used to analyze genomic DNA that was isolated from *P. torridus* cultures as described above. For this, 1 μg genomic DNA was digested with EcoRI and then denatured with 0.3 N sodium hydroxide for 30 min. This was followed by neutralization with 1 M ammonium acetate before spotting on Hybond N^+^ nylon membrane (GE Healthcare, United States) using the Bio-Dot apparatus (Bio-Rad Laboratories, United States). The membrane was baked at 80°C for 2 h before probing with anti-m6A antibody (Sigma Aldrich, Cat. No. ABE572) or anti-m5C antibody (EpiGentek, Cat.No. A-1014-010). This was done by blocking the baked dot blots with 10% skim milk (Difco Laboratories) for an hour at room temperature, washing the blots twice with 1X PBS-T, and then incubating them overnight at 4°C with the anti-m6A or anti-m5C antibodies (1:1000 dil in 1X PBS). Following three washes with 1X PBS-T the blots were incubated with HRP-labelled secondary antibody (1:10000 dil, Jackson Laboratory, United States) for an hour and developed using a chemiluminescence method.

### 2.7. DNA methylation assay

DNA methylation assays were performed using a dot blot-based assay with lambda DNA (as the substrate) that had been isolated from phage particles hosted by an *E.coli dam^−^dcm^−^* strain (DNA purchased from Thermo, United States; Cat no: SD0021). Reactions (10 μl) were typically carried out in a Tris-acetate based buffer [30 mM Tris-acetate (pH 5.0), 10 mM potassium acetate, 10 mM magnesium acetate, 100 μg/ml BSA] containing 100 μM S-adenosyl methionine (AdoMet; purchased from NEB, United States), 12 mM 2-mercaptoethanol, 20 mM sodium chloride, 1 μg DNA and 1.4 μM M.PtoI, at 55°C for 1 h. Reactions were stopped with 100 mM EDTA. Reactions were analyzed by dot blot assay as described above. The blots were quantified using ImageJ analysis, and the ratios of m6A with reference to *E.coli* genomic DNA spotted on the same blot was determined. Each experiment was done thrice. The graphs depict average values of three experiments with error bars representing standard deviation.

### 2.8. CD spectroscopy analysis

CD spectroscopy was carried out as earlier (Arora et al., 2014). Briefly, proteins (100 μg in 10 mM potassium phosphate buffer (pH 5.8)) were analyzed with a Jasco J-815 spectropolarimeter using cuvette of 0.1 cm path length. CD spectra were recorded over 250–190 nm at room temperature, in 1 nm steps at a scan speed of 200 nm/min. The presented spectra are the average of 20 scans. The data is depicted as the mean residue ellipticity (MRE).

### 2.9. Homology modelling and structural analysis

The structures of the M and S subunits of M.PtoI were predicted using the Phyre2 (*p*rotein *h*omology/analog*y r*ecognition *e*ngine version 2.0) online server (Kelley et al., 2015).^1^ The reliability of the predicted models were evaluated using the QMean and LG scores obtained (Benkert et al., 2009).^2^ In both cases the models that were considered demonstrated high coverage against their respective templates. All structures were visualized and illustrations created using PyMOL^3^ (Mooers, 2016).

## 3. Results

### 3.1. *Picrophilus torridus* genome carries methylated adenine residues

**Figure 1:**
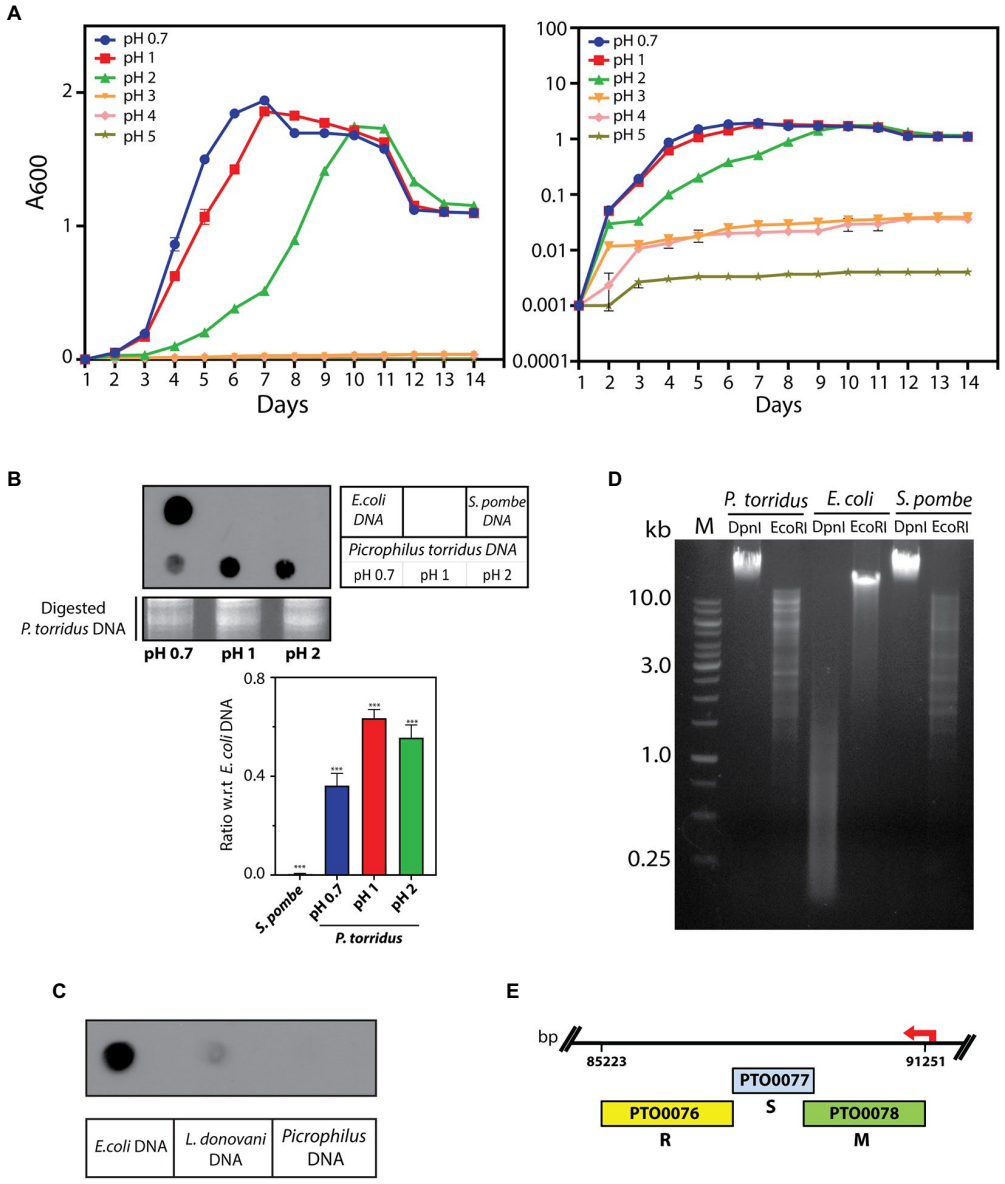
**(A)** Analysis of growth of *P. torridus* at different pH. Cultures were initiated with 1% (v/v) inoculum from a starter culture at pH 1.0 that was at OD_600_ ~ 1.5. Growth was monitored every 24 h. Graph represents average values of three experiments and error bars represent standard deviation. Left panel: data plotted against linear y-axis. Right panel: data plotted against log y-axis. **(B)** Dot blot analysis of *P. torridus* genomic DNA for m6A mark. 1 μg of genomic DNA was used in each case. Upper left panel: blot probed with anti-m6A antibody (1:1000 dil). Loading control: agarose gel analysis of input genomic DNA digested with EcoRI. Upper right panel: loading scheme of dot blot. Lower panel: quantification of *P. torridus* genomic DNA signal as a ratio of *E.coli* genomic DNA signal, done using ImageJ software. Bars represent average of three experiments. Error bars represent standard deviation. Student’s two-tailed *t-*test was applied, ***p < 0.0005. **(C)** Dot blot analysis of *P. torridus* genomic DNA for m5C mark. 1 μg of genomic DNA was used in each case. Upper panel: blot probed with anti-m5C antibody (1:1000 dil). Lower panel: loading scheme of dot blot. **(D)** Analysis of 5′-GA^m6^TC in *P. torridus* genomic DNA. In each case, 1 μg of genomic DNA was treated with restriction enzyme. M: molecular weight marker. **(E)** Line diagram of *P. torridus* Type I RM system: PTO0076: gene for R subunit, PTO0077: gene for S subunit, PTO0078: gene for M subunit.

The study was initiated with examining the growth pattern of the organism in media of different pH. The data obtained revealed that while *P. torridus* grew comparably at pH 0.7 and 1.0, growth was considerably slower at pH 2.0 and no growth was discernible at pH 3–5 (Figure 1A). To determine if DNA methylation exists in this microorganism under the extreme conditions in which it thrives, genomic DNA was isolated from *P. torridus* cells grown in media whose pH ranged over 0.7 to 2 and the DNA analyzed by dot blot assay as described above. The results obtained clearly demonstrated that the *Picrophilus torridus* genome carries methylated adenine residues (m6A) regardless of the pH at which it is growing, although displaying lower levels of methylation at pH 0.7 (Figure 1B). No m5C methylation was detectable (Figure 1C), and we were unable to assess m4C methylation due to lack of quality antibodies.

A previous study by Koike et al. (2005) had established the presence of the *dam* gene in several archaea including *P. torridus* by computational analysis, but Dam-mediated adenine methylation was not experimentally verified in *P. torridus*. To check the methylation status of the *P. torridus* genome at Dam target sites (5′-GATC-3′) we exploited the property of the DpnI restriction enzyme that cleaves methylated but not unmethylated GATC sites. Genomic DNA isolated from *Picrophilus* cells grown in medium of pH 1.0 was thus subjected to DpnI digestion, and it was observed that while *E.coli* genomic DNA was cleaved by the enzyme the *P. torridus* genomic DNA was not (Figure 1D), nor was *S.pombe* genomic DNA (which lacks m6A methylation as evident from Figure 1B). These data indicate that while the *P. torridus* genome carries the m6A mark the organism does not harbor Dam-mediated adenine methylation.

An examination of the *P. torridus* genome sequence (KEGG database)^4^ revealed that in addition to the *dam* gene, the euryarchaeon has genes encoding a Type II R-M system and a Type I R-M system. All Type I modification methylases characterized to date are m6A methylases, and thus we investigated the modification methylase of the *P. torridus* Type I R-M system. The three genes of the *P. torridus* Type I R-M system encoding for R, M and S subunits lie in a single operon on the lower strand of the circular genome (Figure 1E). The investigation of the modification methylase (henceforth referred to as M.PtoI) commenced with cloning the genes encoding M and S subunits.

### 3.2. M.PtoI carries all the motifs that typify Type I modification methylases

**Figure 2:**
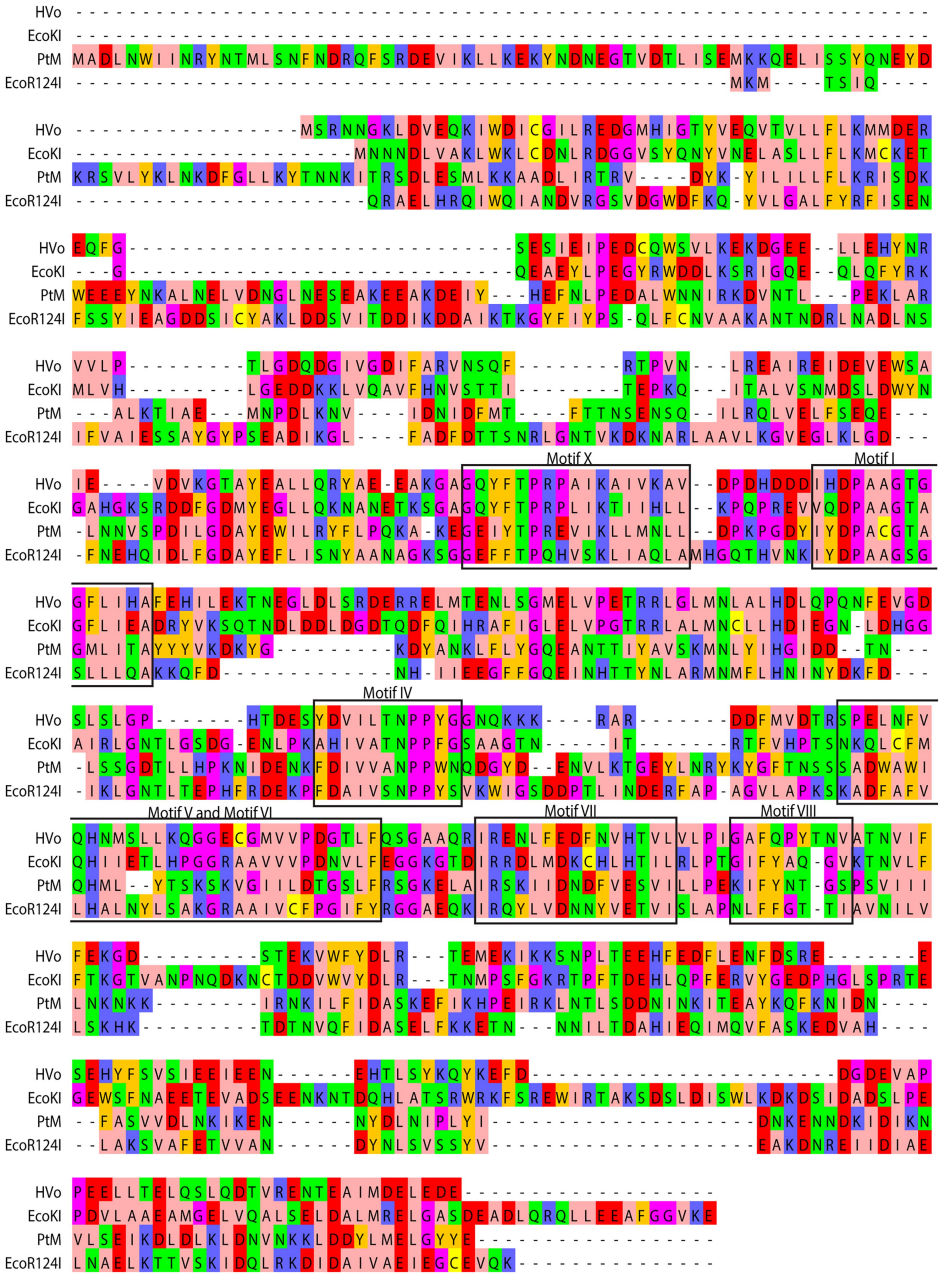
Comparison of derived amino acid sequence of M subunit of M.PtoI with the sequences of M subunits of other Type I modification methylases: Clustal Omega analysis viewed using Jalview multiple alignment editor (Sievers et al., 2011). The conserved motifs are demarcated in black rectangular boxes. Colors are indicative of the physicochemical properties of the amino acids. Pink, aliphatic/hydrophobic; orange/ochre, aromatic; purple, glycine/proline; dark blue, basic; green, hydrophilic; red, acidic; yellow, cysteine.

The M.PtoI genes (designated herewith as PtM and PtS for M and S subunit genes respectively) were cloned by amplification using primers designed against the published PTO0078 and PTO0077 sequences (encoding M and S subunits respectively), as described in Materials and Methods. The pUC clones obtained were sequenced for verification. The sequences of the M and S subunits of M.PtoI were compared with those of typical Type I modification methylases EcoKI and EcoR124I as well as the identified archaeal Hvo MTase (HVO_2270–71), using Clustal Omega and BLASTp analyses (Sievers et al., 2011).^5^ While the M subunit shared ~25–28% sequence identity over a coverage of 55–85% with the M subunits of the other enzymes (Figure 2), the S subunit shared between 23–25% identity over a coverage of 60–93% with their corresponding S subunits (Supplementary Figure S1). A comparison of the sequence of M subunit with the sequences of modification methylases of typical Type II systems using the same tools revealed that although all of them harbor the characteristic motifs discussed below (Supplementary Figure S2), overall there was no significant sequence identity (data not shown), reflecting the differences in the structure and subunit composition of Type I and Type II modification methylases.

m6A and m4C methylases are typified by the presence of nine conserved motifs, identified more than 25 years ago by analyzing the sequences of over 40 DNA MTases (Malone et al., 1995). By examining these motifs in the context of published structures of MTases it is now known that Motifs I-III and X are involved in the binding of the methyl group donor S-adenosyl methionine (AdoMet). It has been suggested that Motif I and motif X form a pocket that lodges the methionine group of AdoMet while motifs II and III are involved in interactions with the ribose and adenine moieties of the same. On the basis of structural as well as biochemical analyses it is also understood that Motifs IV-VIII are part of the active site pocket and are involved in catalysis of the methyl-transfer reaction, while the Target Recognition Domain (TRD) is responsible for recognition of the specific DNA (Malone et al., 1995; Bheemanaik et al., 2006). In Type I enzymes where DNA recognition is not a part of the functions of the M subunit the TRD lies in the S subunit. The recognition sequence of Type I enzymes is bipartite, and accordingly, the S subunits harbor two TRD domains. The MTases have been classified into six categories: α, β, γ, δ, ε, and ζ based on the order of these signature motifs in their primary sequence (Bujnicki, 2002). The majority of the m6A and m4C methylases fall in the α, β, and γ categories. All the Type I modification methylases as well as several Type II methylases are γ MTases, whose order of the motifs is X-I-II-III-IV-V-VI-VII-VIII, and M.PtoI conforms to this as seen in Figure 2. No clearcut motif II and motif III were identifiable in M.PtoI.

### 3.3. The structures of the M and S subunits of M.PtoI are broadly conserved with those of other Type I modification methylases

While the structures of the individual subunits of Type I R-M systems have been solved in case of several enzymes, the holoenzyme (M2S1) structures of only two Type I modification methylases are available to date. The EcoKI modification methylase structures (pdb IDs: 2Y7C and 2Y7H) were proposed based on single particle electron microscopy constructions at 18 Å resolution (Kennaway et al., 2009). The more recent EcoR124I modification methylase and restriction enzyme structures were proposed based on single particle cryo-EM at 4.54 Å resolution (Gao et al., 2020). The basic architecture of the M and S subunits of the two MTases and the interfaces of interaction among the different subunits are largely conserved between the two enzymes. The structures of the M and S subunits of M.PtoI were predicted by modelling using Phyre2. The modelled structures were viewed using PyMOL, which allowed us to superimpose the obtained structures on their respective templates to analyze structural alignments.

**Figure 3:**
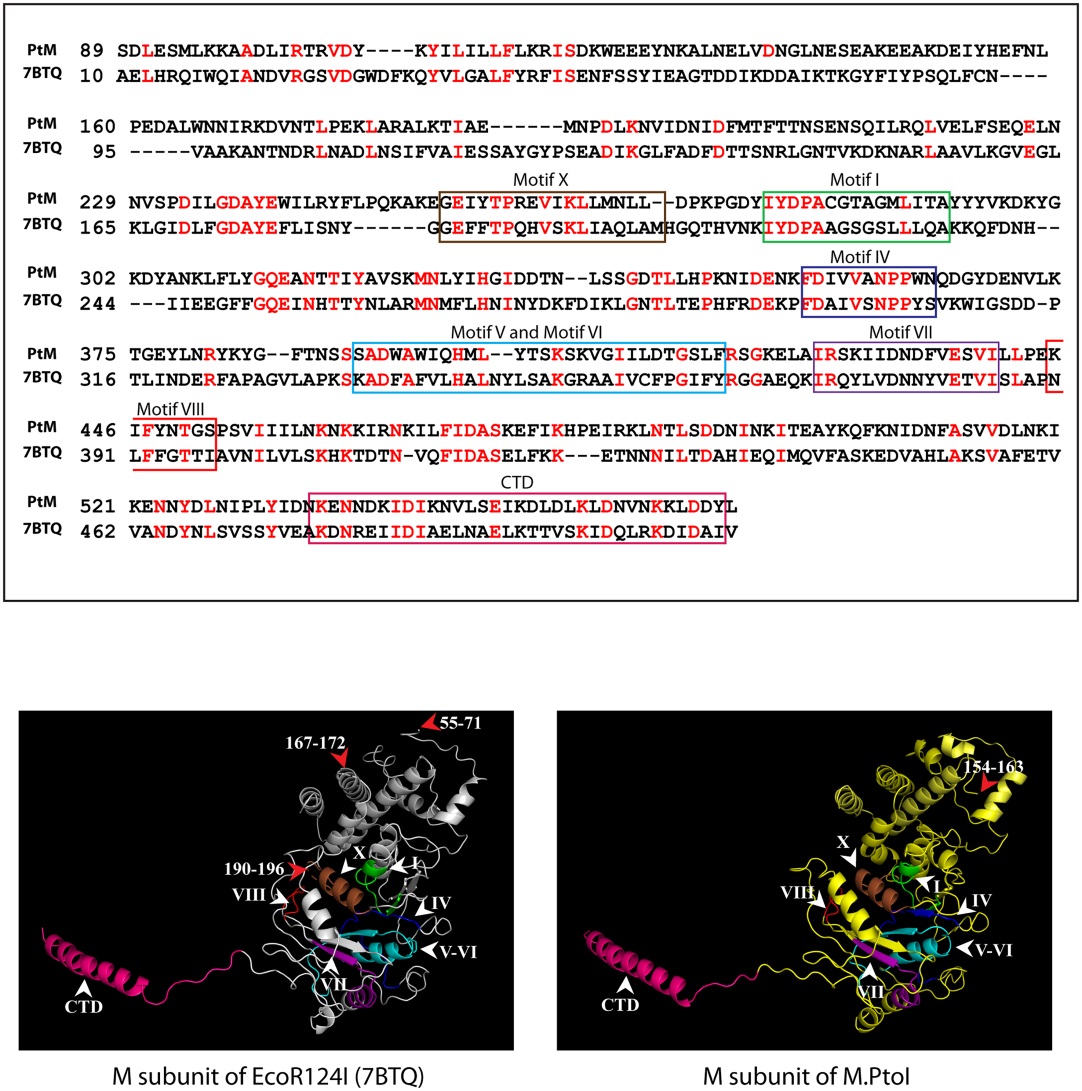
Structural analysis of M subunit of M.PtoI: Upper panel: Alignment of sequence of M subunit of M.PtoI with M subunit of EcoR124I, using Phyre2. The boxes demarcate the positions of the motifs conserved between the two subunits. CTD: C-terminal domain. Lower left panel: Ribbon representation derived from cryo-EM structure (4.54 Å) of EcoR124I [pdb ID: 7BTQ reported by Gao et al. (2020)]. Lower right panel: Ribbon representation of 3D model of M subunit of M.PtoI, modelled against the M subunit of EcoR124I (7BTQ) as template using Phyre2. CTD: C-terminal domain. Roman numerals indicate motif positions. Arabic numerals depict amino acid residues that were excluded from the models.

The most reliable model obtained for the M subunit used the EcoR124I enzyme as template (pdb ID: 7BTQ; Gao et al., 2020). The predicted structure of the M subunit displayed 27% identity with the structure of the M subunits in the 7BTQ cryo-EM structure (4.54 Å), over a coverage of ~83% (residues 89–153 and 164–569). As seen in Figure 3, seven motifs that were aligned in the primary sequence (Figure 2) were also aligned structurally. The M subunits of Type I RM systems are typified by a C-terminal helix that is involved in interactions with the S subunit, and the M subunit of M.PtoI too harbored this conserved helical C-terminal domain (Figure 3).

**Figure 4:**
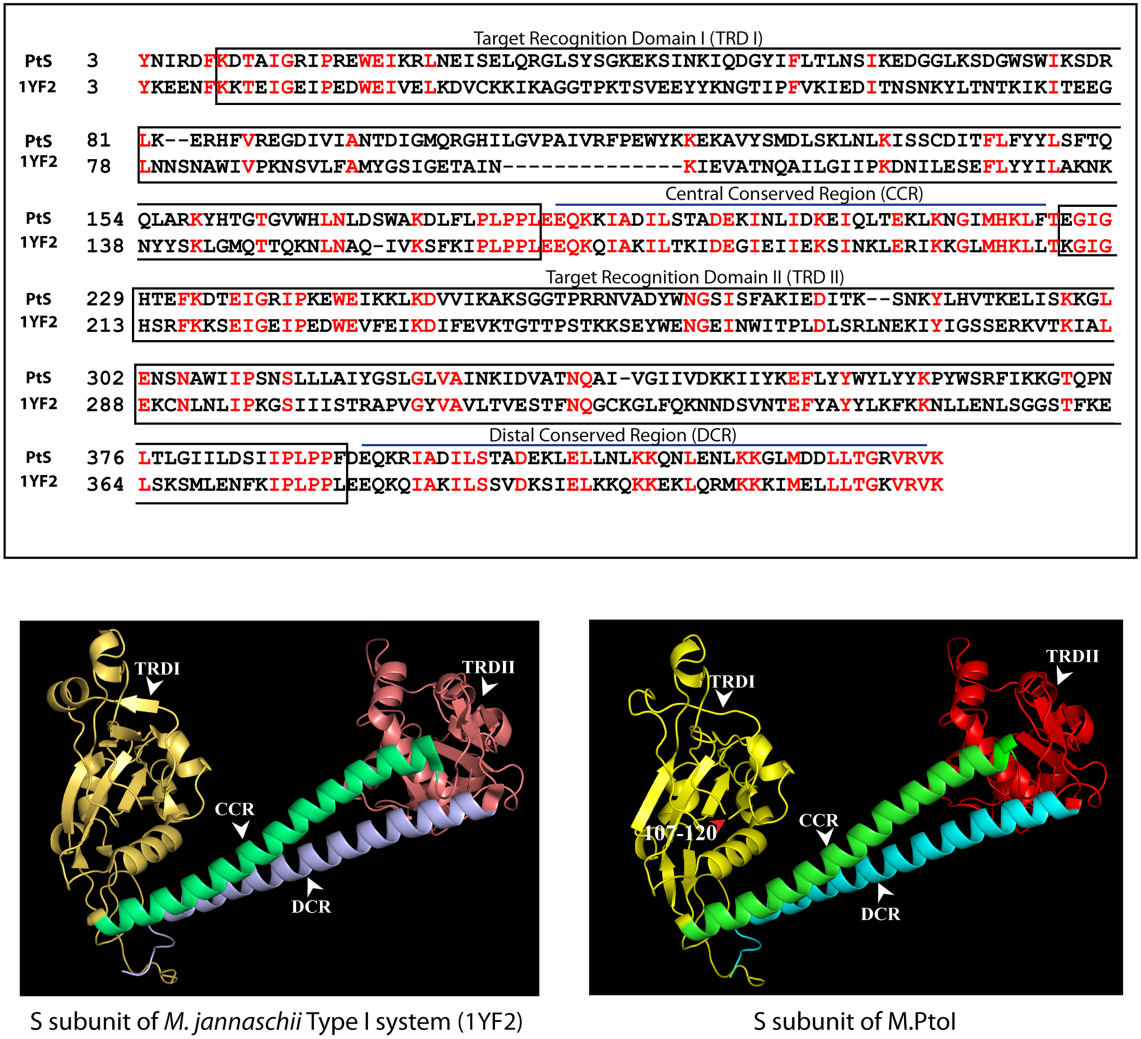
Structural analysis of S subunit of M.PtoI: Upper panel: Alignment of sequence of S subunit of M.PtoI with S subunit of *Methanocaldococcus jannaschii* Type I R-M system, using Phyre2. The boxes demarcate the positions of the domains conserved between the two subunits. Lower left panel: Ribbon representation derived from X-ray crystallography structure (2.4 Å) of *M. jannaschii* Type I RM system’s S subunit [pdb ID: 1YF2, reported by Kim et al. (2005)]. Lower right panel: Ribbon representation of 3D model of S subunit of M.PtoI, modelled against the 1YF2 S subunit structure using Phyre2. Arabic numerals depict amino acid residues that were excluded from the models. TRDI and TRDII: Target Recognition Domain I and Target Recognition Domain II representing the globular domains of the N-terminal and C-terminal TRDs, respectively. CCR: Central Conserved Region, DCR: Distal Conserved Region.

The most reliable structure obtained for the S subunit used the S subunit of the *Methanocaldococcus jannaschii* Type I R-M system (pdb ID: 1YF2; Kim et al., 2005) as template. The S subunit of M.PtoI showed 30% identity with the 1YF2 X-ray crystal structure of the *M. jannaschii* S subunit (2.4 Å), over ~97% coverage (amino acids 6–106 and 121–437). The S subunit of Type I systems harbors two TRDs (target recognition domains) that are oriented inverted with respect to each other. Each TRD carries a globular domain and an alpha-helical dimerization domain (Loenen et al., 2014a). The globular domains (TRDI and TRDII in Figure 4) are responsible for DNA binding, with the N-terminal one binding to the 5′ segment (specific sequence) of the bipartite recognition site and the C-terminal one binding to the 3′ segment (specific sequence) of the same. The alpha-helical domains of the two TRDs (named central conserved region or CCR, and distal conserved region or DCR; see Figure 4) associate with each other such that the two globular domains are held apart at a fixed distance, thus fixing the length of the non-specific spacer sequence in the bipartite recognition site. The architecture of the S subunit of M.PtoI was predicted to be similar, with two TRDs each harboring a globular domain and an alpha helical domain (Figure 4).

**Figure 5:**
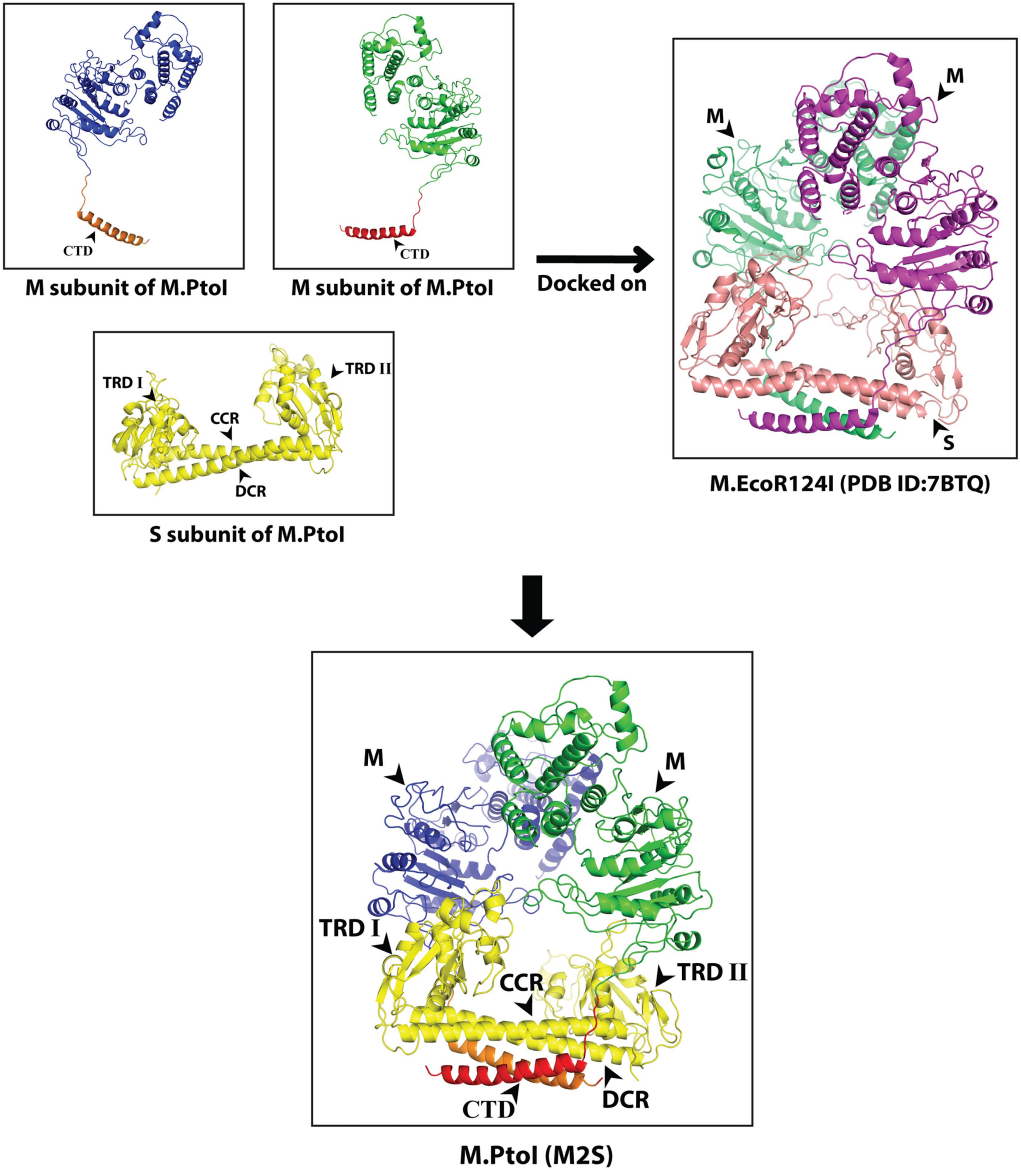
Predicted structure of M.PtoI: Predicted 3D structures of M and S subunits of M.PtoI were superimposed on the 7BTQ M.EcoR124I holoenzyme structure. M.PtoI structure was thus obtained using template-based docking. M: M subunit, CTD: C-terminal domain, TRDI and TRDII: Target Recognition Domain I and II respectively, CCR: Central Conserved Region, DCR: Distal Conserved Region.

The S and M subunits of Type I enzymes characteristically interact with each other (either in the M2S1 or in the R2M2S1 complex) through a four-helix bundle that comprises the C-terminal helical domains of the two M subunits (depicted as CTD in Figure 3) and the two alpha-helical domains of the S subunit (depicted as CCR and DCR in Figure 4). As these regions are conserved in the M and S subunits of M.PtoI, the structure of the M.PtoI holoenzyme (M2S1) was predicted using template-based docking, *via* superimposition of the predicted 3D structures of M and S of M.PtoI (Figures 3, 4) on the M.EcoR124I holoenzyme structure (pdb:7BTQ). The M.PtoI docked structure obtained predicted the two M subunits of the M2S1 holoenzyme to interact with each other *via* their N-terminal domains, and the M and S subunits to interact with each other *via* the four-helix bundle (Figure 5).

**Figure 6:**
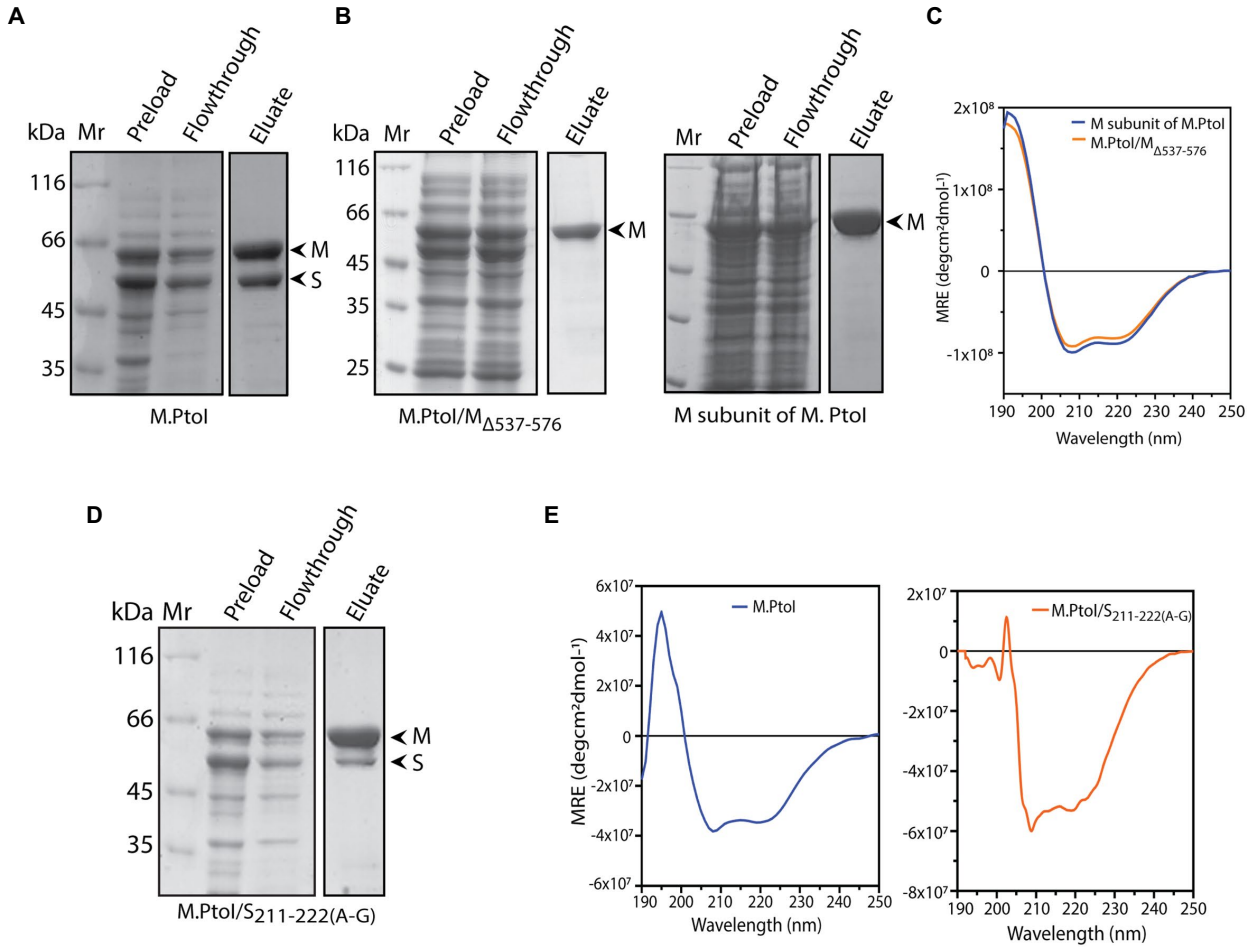
**(A,B,D)** Analysis of purified recombinant M.PtoI proteins. Coomassie-staining of SDS-PAGE. Mr.: molecular weight marker, preload: bacterial whole cell lysates after induction of expression of recombinant proteins, flowthrough: unbound protein fraction after incubation with cobalt affinity beads, eluate: protein fraction after elution with 250 mM imidazole. Arrowheads indicate subunits in eluate fractions. **(A)** Wild type. Eluate: M and S subunits (molecular weights 63 kDa and 48 kDa respectively). **(B)** Left panel: M.PtoI/M_Δ537-576_. Eluate: M_Δ537-576_ subunit only (molecular weights ~58 kDa). Right panel: M subunit of M.PtoI. Eluate: M subunit (molecular weight 63 kDa). **(C)** CD spectra of M.PtoI M subunit and M.PtoI/M_Δ537-576_ depicted as a measure of mean residue ellipticity (MRE). **(D)** M.PtoI/S_211-222(A-G)._ Eluate: M and S subunits (molecular weights 63 kDa and 48 kDa respectively). **(E)** CD spectra of M.PtoI (left panel) and M.PtoI/S_211-222(A-G)_ (right panel) depicted as a measure of mean residue ellipticity (MRE).

The role of the C-terminal helical domain of M in mediating interactions with the S subunit was experimentally validated by creating a C-terminal deletion mutant of M: M.PtoI/M_Δ537-576_, and analyzing it for loss of interactions with S. For this, the recombinant M.PtoI proteins were purified from *E.coli*. Recombinant M.PtoI (full length) was expressed in *E.coli* using the pET-Duet expression vector which allows the simultaneous expression of two proteins from two separate T7*lac* promoters, with one of the two proteins being expressed in fusion with the His tag at the N-terminus. The M and S subunits were thus simultaneously expressed in BL21 Codon Plus *E. coli* cells from the pETDuet/PtSM clone (as described in Methods) such that M was His-tagged. The cell lysates were subjected to cobalt affinity-based chromatography. SDS-PAGE analysis revealed the eluate to carry both subunits in stoichiometric amounts, leading us to conclude that the M.PtoI holoenzyme (M2S1) assembles in the host bacterium, allowing the S subunit to co-purify with the His-tagged M (Figure 6A). In creating the C-terminal deletion of M, amino acids 537–576 were excluded (mutagenesis detailed in Methods) and the M_Δ537-576_ co-expressed with S in BL21 Codon Plus cells from the pETDuet/PtSM_Δ537-576_ clone. When these cell lysates were put through TALON beads the eluate carried only the truncated M subunit, clearly demonstrating loss of interaction with S in M.PtoI/M_Δ537-576_ (Figure 6B, left panel, fourth lane). The deletion did not cause any gross structural changes in the M subunit based on a comparison of the CD spectroscopy profiles of purified M (Figure 6B, right panel, fourth lane) and M.PtoI/M_Δ537-576_ (Figure 6C). Taken together, these results indicate that the C-terminal helical domain of M is functionally conserved in M.PtoI.

The role of the central helical region of the S subunit in mediating M-S interactions was examined by creating a mutant S subunit where 12 amino acids of the central conserved region (CCR) were replaced by a 12 amino acid stretch of alternate glycine and alanine residues to create M.PtoI/S_211-222(A-G)_ (described in “Methods”). When the recombinant M.PtoI/S_211-222(A-G)_ protein was purified from *E.coli* using cobalt affinity chromatography it was found that there was a substantial increase in the ratio of M:S in the eluate fractions (Figure 6D, fourth lane), implying that the replaced region played a significant role in mediating M-S interactions. The difference in CD spectroscopy profiles of the wild type and mutant M.PtoI proteins most likely reflects the differences in M:S stoichiometry in the two proteins (Figure 6E).

M-S interactions in wild type and mutant M.PtoI proteins were also checked using purified proteins. For this, M and S subunits were separately expressed in *E.coli* cells from respective pET/Duet clones carrying either M or S genes. As all M subunits were tagged with His at their N-terminus, they were immobilized on Co-affinity resin. Whole cell lysates harboring recombinant S subunit (wild type or mutant) were added to the reaction, and after washing off the unbound fractions, the bound fractions (M subunits plus any interacting S subunits) were eluted using imidazole and analyzed. As seen in Supplementary Figure S3, while S subunit interacts with wild type M subunit (lane 5), it fails to interact with mutant M_Δ537-576_ subunit (lane 6). The mutant S_211-222(A-G)_ subunit interacts with M subunit to a significantly lesser extent than wild type S (compare lane 7 with lane 5). These data confirm the role of the C-terminus of M subunit and residues 211–222 of S subunit in maintaining M-S interactions.

### 3.4. M.PtoI methylates DNA at adenine residues *in vitro*

**Figure 7:**
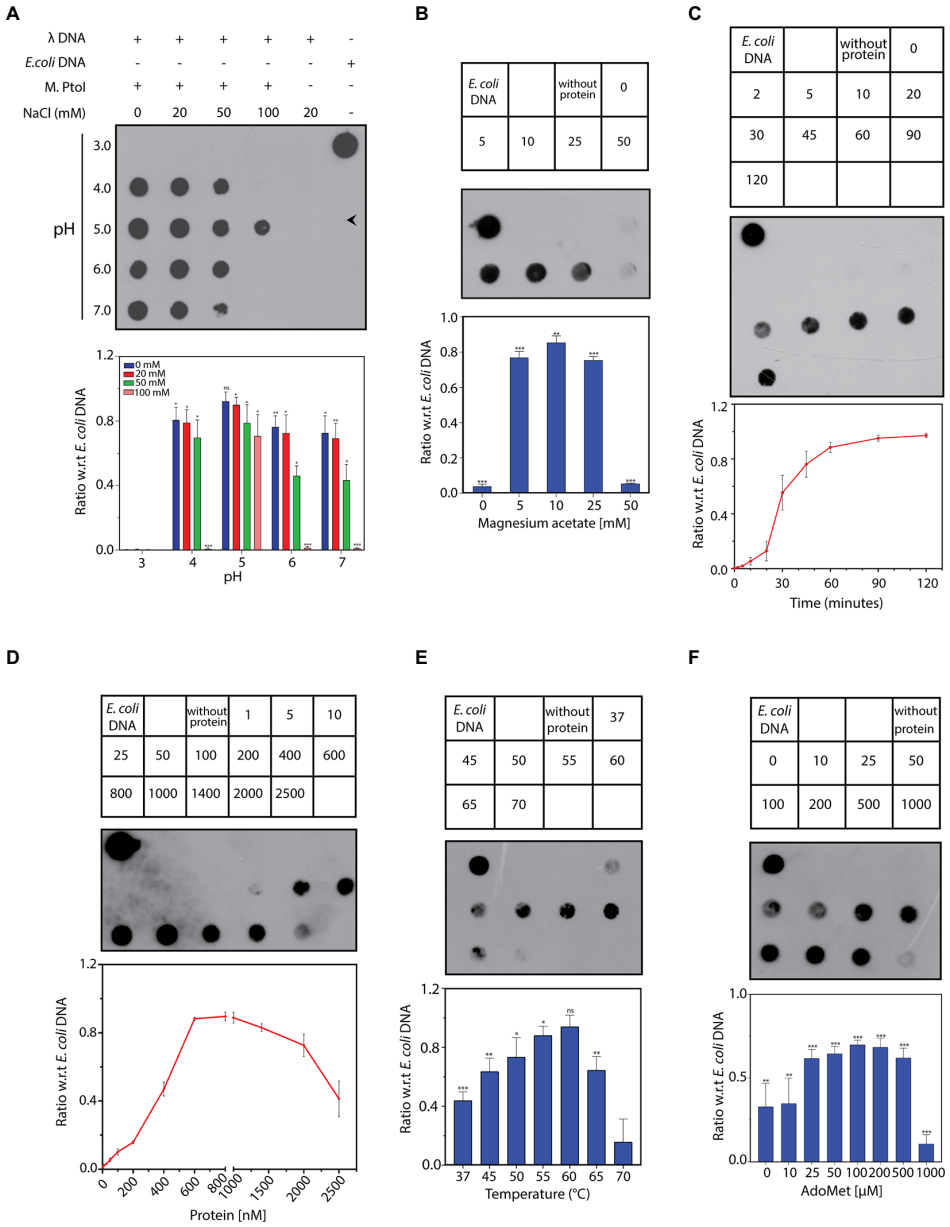
**(A–F)** Dot blot analyses of methylation assays performed with M.PtoI. Assays were performed with 1 μg lambda DNA substrate, in Tris-acetate based buffer (pH 5) carrying 10 mM magnesium acetate, 100 μM S-adenosyl methionine (AdoMet), 20 mM sodium chloride, and 1.4 μM M.PtoI, at 55°C, unless otherwise specified. 1 μg *E.coli* genomic DNA was used as control on every dot blot. Upper panels in each figure: loading scheme of dot blot. Middle panel: Dot blot of assay. Lower panel: quantification of signals from methylation reactions as a ratio of *E.coli* genomic DNA signal, done using ImageJ software. Data represent average of three experiments. Error bars represent standard deviation. Student’s two-tailed *t-*test was applied, *p < 0.05, **p < 0.005, ***p < 0.0005, ns-not significant. **(A)** Reaction as a function of pH and salt concentration: Activity was assayed over pH 3–7 in presence of 0–100 mM sodium chloride, using 1.4 μM M.PtoI at 55°C for 1 h. **(B)** Reaction as a function of magnesium ion concentration: Activity was assayed over 0–50 mM magnesium acetate, using 1.4 μM M.PtoI at 55°C for 1 h. Numbers in the boxes above the blot represent magnesium acetate concentrations (mM). Reaction without protein had 10 mM magnesium acetate. **(C)** Progress curve of the reaction: Activity was monitored over 2–120 min using 1.4 μM M.PtoI, at 55°C. Numbers in the boxes above the blot represent incubation time (min). Reaction without protein was incubated for 120 min. **(D)** Reaction as a function of enzyme concentration: Activity was assayed with 1–2,500 nM M.PtoI, at 55°C for 45 min. Numbers in the boxes above the blot represent enzyme concentrations (nM). **(E)** Reaction as a function of temperature: Activity was assayed over 37–70° C, using 600 nM M.PtoI for 45 min. Numbers in the boxes above the blot represent reaction temperature. Reaction without protein was incubated at 55°C. **(F)** Reaction as a function of AdoMet concentration: Activity was assayed over 0–1,000 μM S-adenosyl methionine (AdoMet), using 400 nM M.PtoI at 55°C for 1 h. Numbers in the boxes above the blot represent AdoMet concentrations (μM). Reaction without protein had 100 μM AdoMet.

The ability of the purified M.PtoI to methylate DNA at adenine residues was analyzed using unmethylated lambda DNA isolated from phage that had been grown in a *dam^−^dcm^−^ E.coli* strain. The reactions were analyzed in dot blot assays using anti-m6A antibody, as described in Methods. Activity assays were initiated at 55°C for 1 h in Tris-acetate buffers whose pH ranged over 3 to 7, in the presence of sodium chloride ranging over 0–100 mM. As seen in Figure 7A M.PtoI was able to methylate lambda DNA (producing m6A) at pH ranging from 4 to 7, with optimum activity being detected at pH 5, which is near the organism’s intracellular pH of 4.6. Higher salt concentrations were detrimental to activity, with optimal activity seen at 0–20 mM NaCl. Thus, all further assays were carried out in Tris-acetate buffer (pH 5) containing 20 mM NaCl. The optimal concentration of magnesium ions that supported the DNA methylation reaction was determined over 0–50 mM magnesium acetate. It was found that while magnesium was necessary for the methylation reaction, concentrations of magnesium over 5–25 mM supported the reaction more or less equivalently, with a higher concentration of 50 mM being inhibitory (Figure 7B). The methylation reaction displayed an initial lag before proceeding almost linearly for approximately the first 45 min and plateaued beyond 60 min (Figure 7C). The ability of M.PtoI to methylate DNA was concentration-dependent, increasing almost linearly between 200 and 600 nM before plateauing (Figure 7D). High concentrations of enzyme (2,000 nM and beyond) proved to be detrimental, perhaps due to protein aggregation occurring at such high concentrations. In carrying out the assays at different temperatures ranging over 37–70°C it was found that M.PtoI was active at temperatures between 37–65°C with maximal activity at 55 and 60°C, in keeping with the conditions in which it thrives (Figure 7E). AdoMet supported the methylation reaction over concentrations varying from 10–500 μM, with 50–100 μM being optimal (Figure 7F). Interestingly, the enzyme displayed substrate inhibition at higher AdoMet concentration of 1 mM. Furthermore, the enzyme was able to methylate DNA, although to a much lesser extent, even in the absence of exogenously added AdoMet, suggesting that endogenous AdoMet remains bound to the M subunit through the purification process. This is not unusual for such enzymes, with similar findings in multiple enzymes such as in BpmI, BseMII, and EcoR124II (Dreier et al., 1996; Jurenaite-Urbanaviciene et al., 2001; Bath et al., 2002). Unsurprisingly, the addition of ATP had no effect on methylation activity (data not shown). Taken together, the results presented in Figure 7 establish M.PtoI as an active m6A MTase.

### 3.5. The NPPW and xxGxxG motifs in M.PtoI are critical for methylation activity

**Figure 8:**
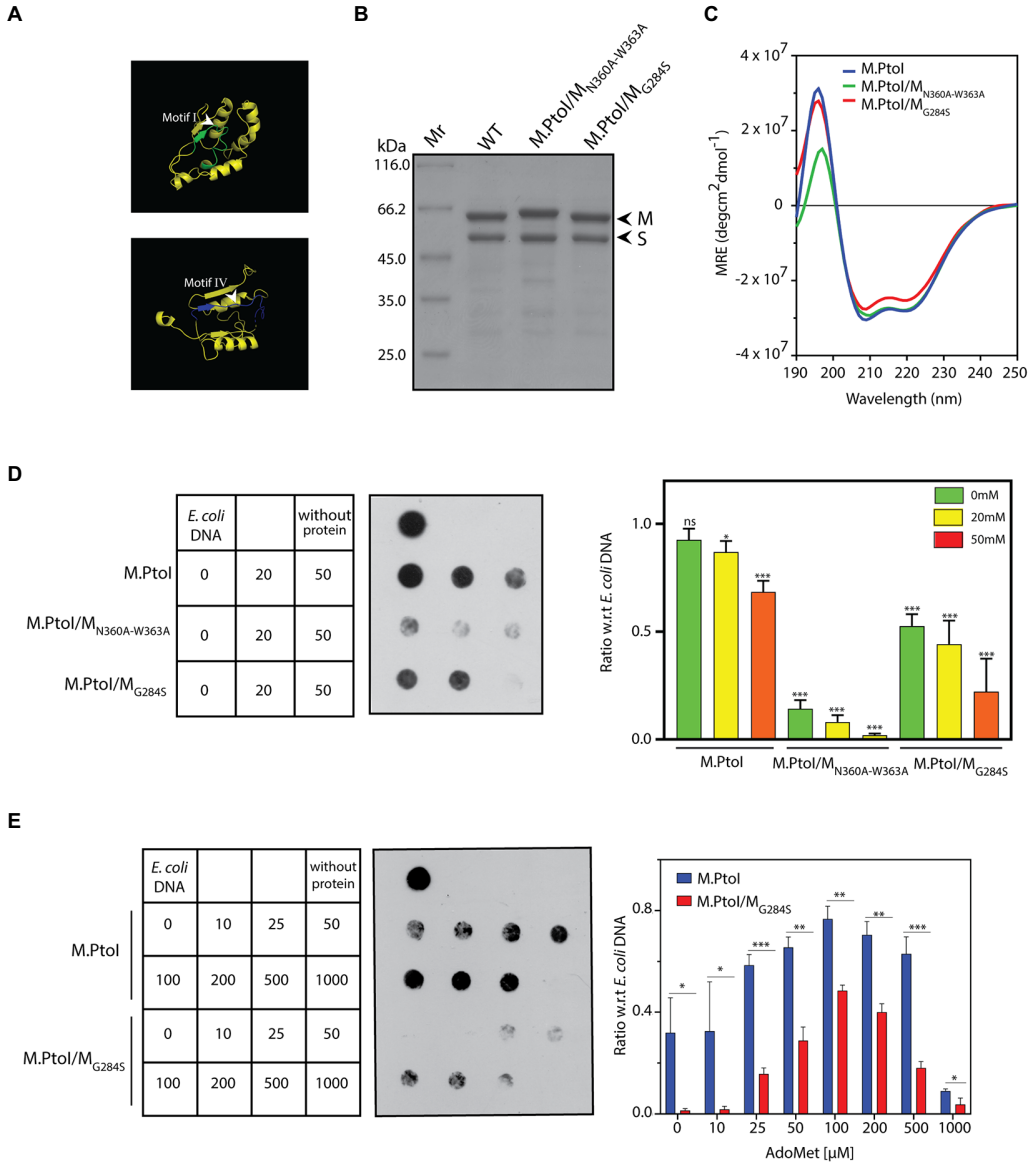
**(A)** Ribbon representation of motif I and motif IV derived from the predicted structure of M subunit of M.PtoI. **(B)** Coomassie stained SDS-PAGE analysis of purified recombinant M.PtoI/M_N360A-W363A_ and M.PtoI/M_G284S._ Mr.: molecular weight marker. Arrowheads depict M and S subunits (63 kDa and 48 kDa respectively). **(C)** CD spectra of M.PtoI/M_N360A-W363A_ and M.PtoI/M_G284S_ depicted as a measure of mean residue ellipticity (MRE). **(D,E)** Dot blot analyses of methylation assays with mutant M.PtoI proteins. Assays were performed as described in the legend to Figure 7 unless otherwise specified. Left panel: loading scheme of dot blot. Middle panel: Dot blot of assay. Right panel: quantification of signals from methylation reactions as a ratio of *E.coli* genomic DNA signal, done using ImageJ software. Data represent average of three experiments. Error bars represent standard deviation. Student’s two-tailed *t-*test was applied, *p < 0.05, **p < 0.005, ***p < 0.0005, ns-not significant. **(D)** Reactions with M.PtoI, M.PtoI/M_N360A-W363A_ and M.PtoI/M_G284S_. Activity assayed as a function of sodium chloride concentration (0–50 mM) at pH 5. Numbers in boxes represent NaCl concentrations (mM). Reaction without protein had 20 mM NaCl. **(E)** Reactions with M.PtoI and M.PtoI/M_G284S_. Activity assayed as a function of AdoMet concentration (0–1,000 μM). Numbers in boxes represent AdoMet concentrations (μM). Reaction without protein had 100 μM AdoMet.

Type I modification methylases, like most adenine MTases, are typified by two primary domains they harbor in their M subunits: the AdoMet binding domain (motifs X, I-III in Figures 2, 3), and the catalytic domain that is responsible for the transfer of the methyl group from the donor to the nucleotide which is to be methylated (motifs IV-VIII in Figures 2, 3). Two motifs that are of singular importance are the AdoMet-binding motif I (FxGxxG) and the catalytic motif IV (NPPY/F/W). The catalytic motif IV lies in a pocket on the surface of the M subunit and at the time of methylation the specific adenine residue flips out of the duplex DNA substrate into the pocket where it stacks with the aromatic residue as well as forms hydrogen bonds with the first amino acid of the motif (which could by D, S, or N), stabilizing it for receiving the methyl group which is directly transferred to the target amino group on the adenine (Bheemanaik et al., 2006). Based on the sequence alignment with other Type I MTases (Figure 2), the FxGxxG motif in M is located at residues 282–287 and the NPPW motif lies at residues 360–363 (Figure 8A). The importance of these two motifs for the methyltransferase activity of M.PtoI was assessed by developing mutant enzymes and analyzing their activity. Accordingly, the glycine residue at position 284 was mutated to serine, and the asparagine and tryptophan residues at positions 360 and 363 were mutated to alanine (described in Methods). The mutated M subunits were co-expressed with S subunit in *E.coli* cells from pETDuet/PtSM_G284S_ and pETDuet/PtSM_N360A-W363A_, respectively. The mutant enzymes M.PtoI/M_G284S_ and M.PtoI/M_N360A-W363A_ were purified (Figure 8B) and assessed for any gross structural changes using CD spectroscopy; no gross structural changes were detectable (Figure 8C).

The two mutant enzymes were assessed for their ability to methylate DNA in Tris-acetate buffer at pH 5 over sodium chloride concentrations ranging from 0–50 mM. M.PtoI/M_N360A-W363A_ was found to be virtually inactive under all tested conditions, while M.PtoI/M_G284S_ was less active than wild type enzyme at all tested conditions (Figure 8D). The activity of M.PtoI/M_G284S_ was compared with that of the wild type M.PtoI at different concentrations of AdoMet. It was observed that while the wild type enzyme supported DNA methylation more or less equivalently over 50–500 μM AdoMet, the mutant enzyme while displaying an overall lower activity showed maximal support of DNA methylation at 100–200 μM AdoMet (Figure 8E). These data establish that the NPPW and xxGxxG motifs in M.PtoI are critical to M.PtoI activity.

## 4. Discussion

DNA methylation is widely prevalent in bacteria and archaea, and known to modulate various cellular processes as well as exist as part of the organisms’ defense mechanisms against foreign DNA. *Picrophilus torridus* grows in conditions of extreme pH, thriving at pH 0.7 to 2 (Figure 1A), and to date no other microorganism growing under these extreme conditions has been isolated. We initiated the study with examining whether the *Picrophilus* genome is methylated, and found it to carry the m6A modification mark but not the m5C mark (Figures 1B,C). Interestingly, the extent of adenine methylation was significantly lower when the organism was grown at pH 0.7 as compared to when grown at pH 1 or 2. The commonest mediator of adenine methylation in prokaryotes is the Dam methylase. The *E. coli* Dam methylase regulates the timing of replication origin firing *via* methylation of the GATC sites in the *OriC* region. Dam-mediated GATC methylation also modulates methyl-directed mismatch repair (Adhikari and Curtis, 2016), and has been identified as a regulator of gene expression in *E. coli*, *Yersinia*, and *Salmonella typhimurium* (Falker et al., 2007; Brunet et al., 2020; Keceli Oguz et al., 2022). Widely prevalent across bacteria, some archaea also harbor the Dam. *Halobacterium saccharovorum* and *Methanobacterium* strain Ivanov have been reported to be Dam^+^ as early as 1984 (Barbeyron et al., 1984), and the *in vitro* methylation activity of the Dam methylase of *Pyrococcus horikoshii* has also been examined (Maynard-Smith et al., 2011). Koike et al. (2005) identified the Dam methylase in multiple archaea species through annotation of genome sequences. Their experimental analysis found *Thermoplasma volcanium, Thermoplasma acidophilum*, and *Pyrocococcus* species OT3 genomes to carry methylated 5′-GATC sequences, while *Sulfolobus solfataricus* and *Sulfolobus shibatae* did not show evidence of 5′-GATC methylation. A subsequent study by Couturier and Lindas (2018) identified the methylation of 5′-GATC sites in *Sulfolobus acidocaldarius*. While the Dam methylase was identified by sequence annotation in *P. torridus* in the study by Koike et al. (2005), 5′-GATC methylation was not experimentally verified in that study. Our results indicated lack of 5′-GATC methylation in the *P. torridus* genome (Figure 1D), suggesting the absence of an active Dam in this organism and implicating the possible role of an R-M system in mediating adenine methylation in the organism.

The genes encoding the components of the single Type I R-M system identified through sequence annotation of the *P. torridus* genome lie in a cluster, on the lower strand of the genome between positions 85,223 and 91,251 (Figure 1E), and the modification methylase component of this system, named M.PtoI, was investigated for its ability to methylate adenine residues. The structure of M.PtoI and the interacting interfaces of the M and S subunits were predicted to be conserved with those of other Type I R-M systems (Figures 3–5), and the predictions were experimentally verified in part by the creation and analysis of suitable M and S mutant proteins (Figure 6), which confirmed that the M subunits interacted with the central conserved region (CCR) of the S subunit through their C-terminal helical domains (CTDs). The ability of M.PtoI to methylate DNA was assessed using the recombinant protein in *in vitro* assays. Activity was found to be optimal at pH and temperature conditions that reflected the organism’s lifestyle and physiological state (Figures 7A,E). The M.PabI Type II modification methylase of *Pyrococcus abyssi* has been found to methylate DNA at 95°C as much as at 65°C although optimally at 85°C, and demonstrated optimal activity at pH 5.8 to 6.7, similarly reflecting the growth and intracellular conditions of the hyperthermophilic organism (Watanabe et al., 2006). Magnesium ions were found to be essential for M.PtoI activity (Figure 7B). Magnesium ions (but not calcium or manganese ions) have been found to be essential for M.EcoP15I methyltransferase activity as well. CD spectroscopy analysis of M.EcoP15I titrated against increasing concentrations of magnesium revealed a secondary structure alteration in the enzyme in response to magnesium ions, suggesting that magnesium ion-induced conformational changes preceded the catalysis reaction. Mutation of the magnesium binding motif resulted in enzyme inactivity (Bist and Rao, 2003). Earlier studies with EcoBI as well as MmeI methyltransferases indicated that the presence of magnesium ions, though not essential to the methylation reaction, stimulated it (Lautenberger and Linn, 1972; Tucholski et al., 1995). M.PtoI was found to be active over AdoMet concentrations 25–500 μM, exhibiting substrate inhibition at 1 mM AdoMet. The purified protein most likely harbored endogenous AdoMet as it exhibited methylation activity even when AdoMet was not added to the reaction (Figure 7F), a feature of a few other methyltransferases as well (Dreier et al., 1996; Jurenaite-Urbanaviciene et al., 2001; Bath et al., 2002).

M.PtoI harbored all the motifs typifying adenine methyltransferases (Figure 2). The functional roles of these motifs have been uncovered by a combination of structural and biochemical studies of other DNA methyltransferases. The methylation reaction is mediated by a base-flipping mechanism, wherein the target adenine residue flips out of the double helix into the catalytic pocket (Goedecke et al., 2001). Motif IV (NPPW) is a primary constituent of the catalytic pocket, and the interaction of the flipped target adenine with the asparagine residue is crucial for catalysis. The data from structural and biochemical studies lead us to believe that hydrogen bonding between the N^6^-amino group of the adenine (which serves as the donor) and the asparagine side chain of the NPPW motif possibly polarizes the amino group, promoting the direct transfer of the methyl group from AdoMet to the adenine N^6^ position (Pogolotti et al., 1988; Labahn et al., 1994; Goedecke et al., 2001; Bheemanaik et al., 2006). The tryptophan residue at the last position of the motif can be replaced with any other aromatic residue without any significant impact on catalytic activity, but replacement with other residues has a negative impact on activity (Willcock et al., 1994; Ahmad et al., 1995; Pues et al., 1999). In keeping with these findings, the ability of the M.PtoI/M_N360A-W363A_ motif IV mutant to methylate DNA was severely compromised (Figure 8D). Structural studies of AdoMet-bound enzyme as well as in-depth mutational analyses coupled to biochemical assays have together revealed that motif I plays a major role in AdoMet binding, though it is not directly involved in catalysis *per se* (Labahn et al., 1994; Willcock et al., 1994; Ahmad et al., 1995; Roth et al., 1998; Goedecke et al., 2001). It is possible that the M.PtoI/M_G284A_ mutant displays weaker binding of AdoMet, as the mutant (unlike the wild type enzyme) does not appear to be harboring endogenous AdoMet when purified from *E.coli* since no catalysis is detected at 0 μM AdoMet (Figure 8E). Collectively, the data suggest that the functions of motif I and motif IV may be conserved in M.PtoI.

To date, only one Type I R-M system has been investigated in archaea: that of *Haloferax volcanii.* While several MTases have been annotated in the *Haloferax* genome sequence, a combination of single molecule real time sequencing and gene knockout analyses have demonstrated that only two motifs are methylated in the genome, one of which is methylated at a cytosine residue *via* a Type II enzyme and the second of which (5′-GCAm6BN6VTGC-3′) is methylated by a Type I adenine modification methylase (Ouellette et al., 2015, 2018). The Type I modification methylase harbors all the motifs that typify these enzymes. Interestingly and unusually, adenine methylation was detected only in the first half of the bipartite recognition sequence, suggesting that either the second half of the sequence does not exhibit methylation on the lower strand, or methylation does not occur equivalently on the two strands and thus remains undetected during sequencing.

Taken together, the data presented here establish that even organisms growing in such extreme conditions (as *Picrophilus torridus* does) exhibit DNA methylation. We find evidence for m6A but not m5C methylation in *P. torridus,* and the absence of GATC methylation indicates that the organism lacks an active Dam. Our results suggest that the Type I R-M system annotated in the *P. torridus* genome sequence is active and mediates m6A methylation. We were unable to analyze the *in vivo* role of M.PtoI as *Picrophilus* species are not amenable to genetic manipulations. Attempts to carry out complementation analyses in *E.coli dam*^−^ strain (a kind gift from the Lab Collection of Manjula Reddy at CSIR-CCMB, India) did not succeed as the M and S subunits did not get expressed (from the tetracycline-driven promoter system in pASKIBA43PLUS) in this strain, which is deficient in *argU*, *ileY*, and *leuW* tRNA genes (Supplementary Figure S4). Future studies will be directed toward identifying the target site of M.PtoI and characterizing the PtoI restriction enzyme (M2R2S1).

## Data availability statement

The raw data supporting the conclusions of this article will be made available by the authors, without undue reservation.

## Author contributions

SS and MG designed the project. PG and AS performed the research and prepared the figures. PG, AS, MG, and SS analyzed the data. SS wrote the paper. All authors contributed to the article and approved the submitted version.

## Conflict of interest

The authors declare that the research was conducted in the absence of any commercial or financial relationships that could be construed as a potential conflict of interest.

## Publisher’s note

All claims expressed in this article are solely those of the authors and do not necessarily represent those of their affiliated organizations, or those of the publisher, the editors and the reviewers. Any product that may be evaluated in this article, or claim that may be made by its manufacturer, is not guaranteed or endorsed by the publisher.

## Footnotes

^1^ http://www.sbg.bio.ic.ac.uk/phyre2

^2^ https://proq.bioinfo.se/ProQ/ProQ.html

^3^ http://pymol.org

^4^ www.genome.jp/kegg

^5^ www.blast.ncbi.nlm.nih.gov
