# Supplementary material for: The extremophile Picrophilus torridus carries a DNA adenine methylase M.PtoI that is part of a Type I restriction-modification system
Source: Front Microbiol. 2023 Mar 15;14:1126750. doi: 10.3389/fmicb.2023.1126750 (PMC10050889; doi:10.3389/fmicb.2023.1126750)
Supplement: Supplementary file 1 [file Data_Sheet_1.pdf]

The extremophile *Picrophilus torridus* carries a DNA adenine methylase M.PtoI that is part of a Type I restriction-modification system

*Pallavi Gulati[1], Ashish Singh[1], Manisha Goel[2] and Swati Saha[1]**

[1]Department of Microbiology
University of Delhi South Campus
New Delhi-110021
India

[2]Department of Biophysics
University of Delhi South Campus
New Delhi-110021
India

***To whom correspondence may be addressed
ss5gp@yahoo.co.in, ssaha@south.du.ac.in

Keywords: DNA adenine methylase, Type I restriction-modification system, modification methylase, *Picrophilus torridus*, acidophile, extremophile

Running title: *Picrophilus torridus* Type I modification methylase

Supplementary Figure 1

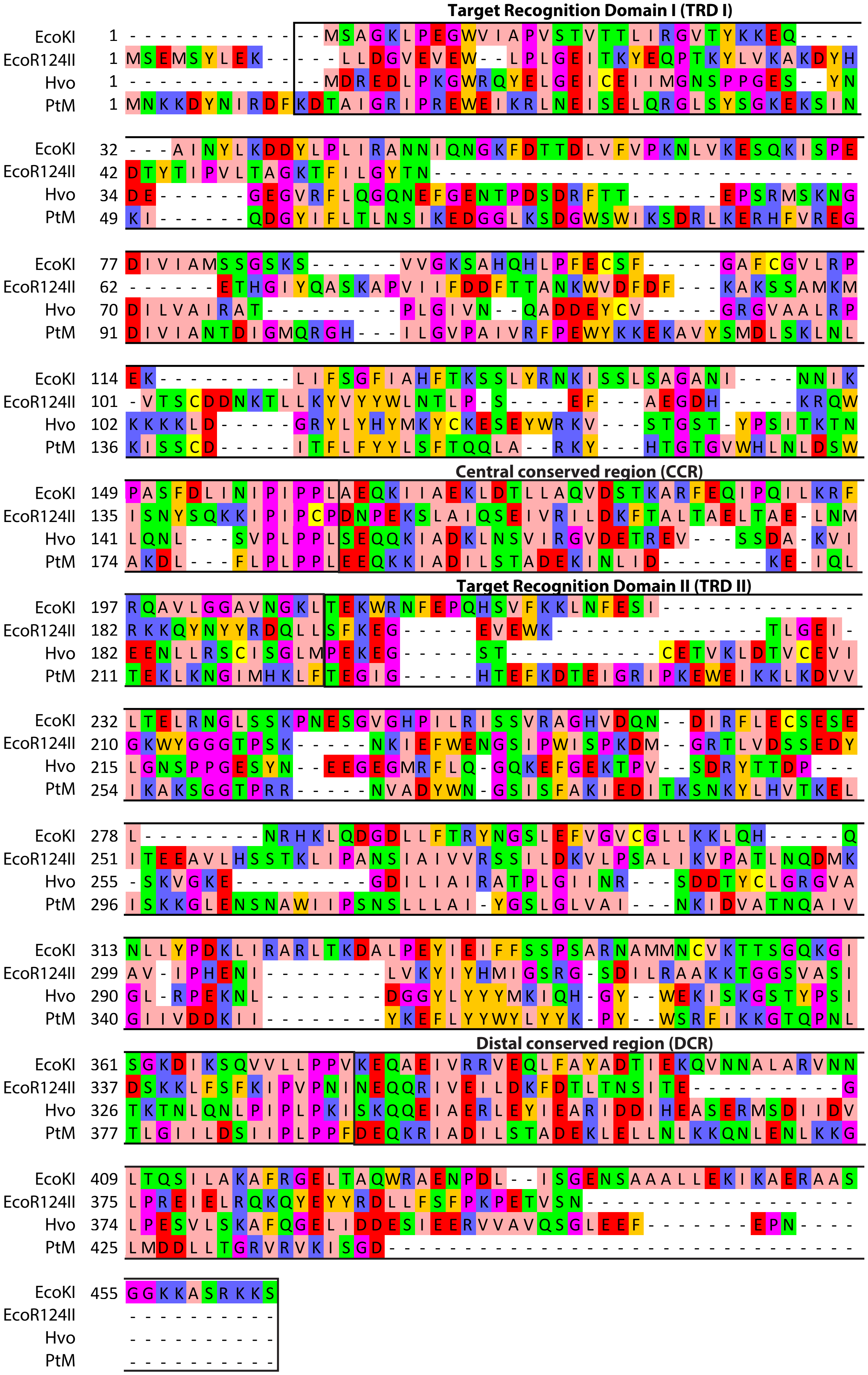

Supplementary Figure 1: Comparison of derived amino acid sequence of S subunit of M.PtoI with the sequences of S subunits of other Type I modification methylases: Clustal Omega analysis viewed using Jalview multiple alignment editor (Sievers et al., 2011). The conserved domains are demarcated in black rectangularboxes. Colors are indicative of the physicochemical properties of the amino acids. Pink,aliphatic/hydrophobic; orange/ochre, aromatic; purple, glycine/proline; dark blue, basic; green,hydrophilic; red, acidic; yellow, cysteine.

Supplementary Figure 2

		Motif X		Motif I		Motif II
PstI	36	GQFMSSSAVSELMANLF	61	ILDAGAGVGSLTAAF	110	KIRAMEFE
TaqI	19	GRVETPPEVVDFMVSLA	43	VLEPACAHGPFLRAF	66	RFVGVEID
EcoRI	50	PRVSNFFKYFAVNFDNL	79	NKEGFSSSEAAKNGF	104	KLVFDDIS
HhaI	298	GNSVVINVLGYIAYNIG	14	FIDLFAGLGGFRLAL	35	CVYSNEWD
PtM	254	GEIYTPREVIKLLMNLL	278	IYDPACGTAGMLITA		

		Motif III		Motif IV
PstI	124	IIESDFIQA	146	YNKAILNPPYLKIAAKGR
TaqI	85	GILADFLLW	99	FDLILGNPPYGIVGEASK
EcoRI	123	SESIDLLKK	133	SDIVVTNPPFSLFREYLD
HhaI	56	KPEGDITQV	72	HDILCAGFPCQAFSISGK
PtM			354	FDIVVANPPWNQDGYD--

		Motif V and VI		Motif VII
PstI	177	NLYSAFVALAI-KGLKSGGELVAITPRSFC	213	FRKQMLDECSLNKIH
TaqI	141	NLYGAFLEKAV-RLLKPGGVLVFVVPATWL	177	LREFLAREGKTSVYY
EcoRI	175	NLIKENKIWLGVHLGR--GVSGFIVPEHYE	208	ARIDSNGNRIISPNN
HhaI	99	TLFFDI-ARIV-REKK--PKVVFMENVKNF	136	VKNTMNELDYSFHAK
PtM	354	SADWAWIQHML--YTSKSKVGIILDTGSLF	426	IRSKIIDNDFVESVI

		Motif VIII
PstI	232	RKSAFKASD
TaqI	192	LGEVFPQKK
EcoRI	228	NLDVFIRHK
HhaI	161	QKRERIYMI
PtM	444	-EKIFYNTG

Supplementary Figure 2: Comparison of derived amino acid sequence of the motifs of M subunit of M.PtoI with the sequences of the motifs of Type II modification methylases.

Supplementary Figure 3

(A)

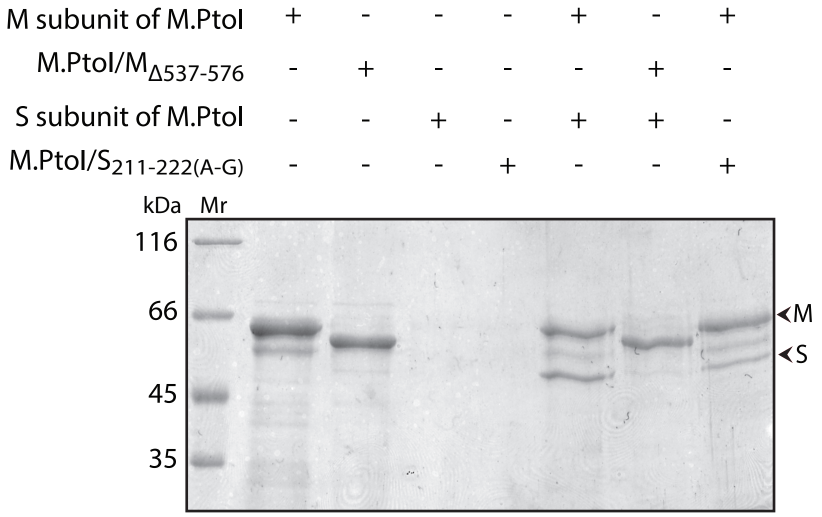

Supplementary Figure 3: Pulldown of S subunit by M subunit using cobalt affinity resin: SDS-PAGE analysis of eluate fractions of pulldown experiment: Coomassie staining. Lane1: wild type M subunit only. Lane 2: truncated M subunit only. Lane 3: whole cell extracts with expressed wild type S subunit only. Lane 4: whole cell extracts with expressed mutant S subunit only. Lane 5: wild type M subunit incubated with wild type S subunit prior to pulldown-elution. Lane 6: truncated M subunit incubated with wild type S subunit prior to pulldown-elution. Lane 7: wild type M subunit incubated with mutant S subunit prior to pull-down-elution. Lane 8: molecular weight marker.

Supplementary Figure 4

(A)

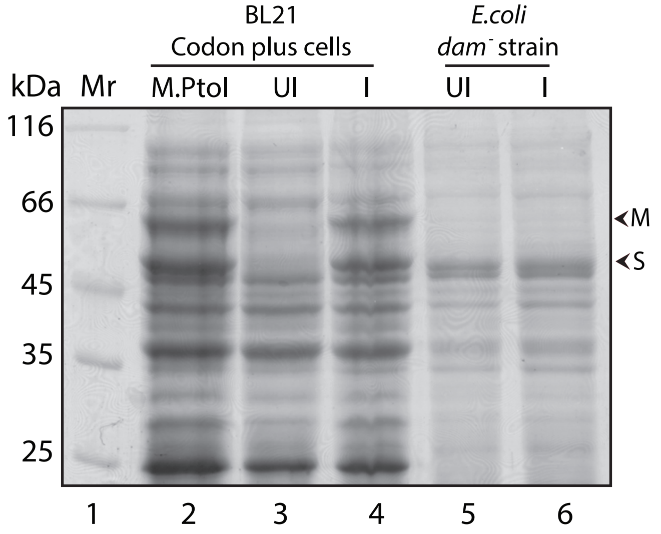

(B)

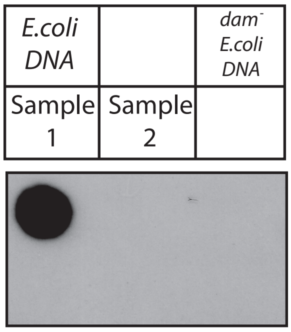

Supplementary Figure 4: A. Coomassie staining of SDS-PAGE analysis of overexpressed M.PtoI. Genes encoding M and S subunits of M.PtoI were cloned into the polylinker of pASKIBA43PLUS under the control of the tet promoter system. The plasmid was transformed into *E.coli* dam^- strain DH5α *dam*$^-$ recA::kan^r as well as BL21 Codon Plus cells, and expression induced in transformant cultures. Induction was carried out for 3 hours prior to harvesting cells and analysis of expression. Lane 1: Molecular weight marker. Lane 2: expression from pET-Duet/PtSM in BL21 Codon Plus cells. Lanes 3 and 4: expression from pASK/PtSM in BL21 CodonPlus cells. UI: uninduced, I: induced. Lanes 5 and 6: expression from pASK/PtSM in *E.coli dam*$^-$ strain DH5α *dam*$^-$ recA::kan^r cells. UI: uninduced, I: induced.

B. Analysis of m6A modification mark in genomic DNA isolated from *E.coli* dam^- strain DH5α dam^- recA::kan^r cells transformed with pASK/ PtSM. Upper panel: loading scheme of dot blot. Lower panel: Dot blot analysis with anti-m6A antibody. Sample 1: genomic DNA isolated from *E.coli* dam^- strain DH5α *dam*$^-$ recA::kan^r transformed with empty pASK vector. Sample 2: genomic DNA isolated from *E.coli dam*$^-$ strain DH5α *dam*$^-$ recA::kan^r transformed with pASK/PtSM followed by induction treatment.
